# Survey of Spanish dentists on the prescription of antibiotics and antiseptics in surgery for impacted lower third molars

**DOI:** 10.4317/medoral.20669

**Published:** 2015-11-30

**Authors:** María-Iciar Arteagoitia, Eva Ramos, Gorka Santamaría, Julio Álvarez, Luis Barbier, Joseba Santamaría

**Affiliations:** 1 MD, DDS, PhD, Professor and Chair, Maxillofacial Surgery Department, BioCruces Health Research Institute, Cruces University Hospital, University of the Basque Country (UPV/EHU), Bizkaia, Spain; Consolidated research group (UPV/EHU IT821-13); 2 MD, DDS, PhD, Associate Professor, Stomatology I Department, University of the Basque Country (UPV/EHU), BioCruces Health Research Institute, Spain; Consolidated research group (UPV/EHU IT821-13); 3 PhD, Degree in Farmacy, BioCruces Health Research Institute, Cruces University Hospital. Spain; 4 DDS, PhD, Associate Professor, Stomatology I Department, University of the Basque Country (UPV/EHU), BioCruces Health Research Institute, Spain; Consolidated research group (UPV/EHU IT821-13); 5 MD, PhD, Associate Professor, Maxillofacial Surgery Department, BioCruces Health Research Institute, Cruces University Hospital, University of the Basque Country, UPV/EHU, Spain; Consolidated research group (UPV/EHU IT821-13); 6 MD PhD, Chair Professor, Maxillofacial Surgery Department, BioCruces Health Research Institute, Cruces University Hospital, University of the Basque Country (UPV/EHU), Spain; Consolidated research group (UPV/EHU IT821-13)

## Abstract

**Background:**

This study explored the attitude of registered dentists in Biscay towards prescribing antibiotics and/or antiseptics to prevent potential infections after surgical extraction of completely bone-impacted third molars in otherwise healthy individuals, with no history of infection.

**Material and Methods:**

We sent letters to 931 registered dentists in Biscay, with an explanation of the study objectives, description of a case of lower third molar impaction, including a panoramic radiograph, and a questionnaire. The questionnaire asked whether they would prescribe antibiotics and/or antiseptics, in the hypothetical case of lower third molar extraction surgery presented, and if so, when, what type, at what dose and how long for.

**Results:**

The questionnaire was completed by 261 dentists (28%), with a mean age of 44.3 years old (SD 11.05) and mean of 18.7 years working as a dentist (SD 9). A total of 216 dentists (82.7%) considered it necessary to prescribe antibiotics. Of these, 126 (58.3%) would prescribe amoxicillin and 74 (34.5%) amoxicillin/clavulanic acid, while 129 dentists (59%) would prescribe antibiotics both before and after surgery and 10 (4.6%) only after surgery. The most common doses were amoxicillin 500 mg or 750 mg every 8 hours, and amoxicillin/clavulanic acid 875/125 mg every 8 hours, in both cases for a mean of 7 days. Further, 74 dentists (28%) said they would use immediate post-extraction socket irrigation with chlorhexidine, while 211 (81%) would prescribe antiseptics in the postoperative period, of whom 97% recommended chlorhexidine. We did not find significant differences in the use of antibiotics or antiseptics by dentist age (ANOVA *p*=0.22 and *p*=0.53, respectively), or professional experience (ANOVA *p*=0.45 and *p*=0.62).

**Conclusions:**

In our sample, the prophylactic prescription of antibiotics and/or chlorhexidine is widespread in clinical practice, in most cases amoxicillin and amoxicillin/clavulanic acid for a week, starting the treatment before surgery.

**Key words:**Extraction, lower third molar, survey, antibiotics, antiseptics.

## Introduction

Lower third molar extraction (TME) is one of the most widely performed procedures in the field of oral surgery. To prevent infectious postoperative inflammation and infection, various different antibiotic regimens are prescribed. Although clinical trials and literature reviews have been conducted on lower TME in healthy patients, there is no consensus on its effectiveness, and hence practice varies between dentists (1-6). On the other hand, well-documented studies report increases in bacterial resistance to antibiotics and underline the need for a rational use of these drugs (7,8). Regarding antiseptics, there is some evidence that the use of antiseptic solutions, for intraoperative irrigation and/or as postoperative antiseptic mouthwashes, to control biofilm after TME may improve outcomes in the postoperative period, reducing the rate of complications (9-13).

In this context, the aim of this study was to explore which prophylactic treatments (antibiotics and/or antiseptics) dentists tend to prescribe in extraction of completely bone-impacted lower third molars, in theory the most difficult to remove. For this, we carried out a survey by sending a questionnaire to 931 dentists in the province of Biscay (Spain), which has a population of over a million.

## Material and Methods

The study was approved by the local research committee and the patient signed an informed consent. The main objective of the survey was to assess whether dentists tend to prescribe antibiotics and/or antiseptics intra- and/or post operatively to prevent potential infections after surgery for the extraction of a completely bone-impacted lower third molar in healthy subjects, with no history of infection, and if so, when, and which agents and dosage would they use.

We designed a short, clear, precise and specific questionnaire that had been piloted previously. It consisted of the following questions. First, there were two open questions concerning their year of birth and the number of years they had worked as a dentist.

**Figure 1:**
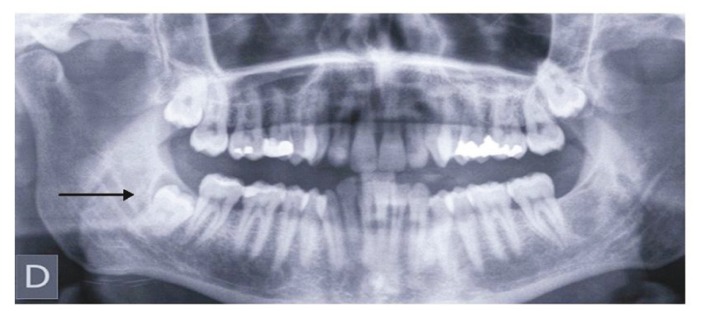
Panoramic radiograph showing an impacted lower third molar completely covered in bone, the tooth indicated for extraction in the case described in the survey.

Second, there was a dichotomous question, “Do you think it would be necessary to prescribe an antibiotic prophylactic ally to prevent a potential infection after the extraction of a completely bone-impacted lower third molar, like the one shown in the panoramic radiograph?” (with response options: Yes or No; ( Fig. 1). If the answer was affirmative, the dentists were asked to respond to the following open questions: “Which antibiotic would you prescribe, when, at what dose and for how long?”

Lastly, the questionnaire contained similar questions regarding the use of antiseptics: namely whether they would perform intraoperative socket irrigation with an antiseptic, and if so, with which one; and whether they would prescribe a postoperative antiseptic, and if so, which one.

The questionnaires were sent out by post. As there was no interviewer present to provide background, we included a cover letter, explaining the objectives of the study, underlining that the research was supported by the College of Pharmacists of Biscay, requesting their participation, and guaranteeing their anonymity. Further, we included a description of the hypothetical case to which the questionnaire referred and a prepaid envelope for returning the completed questionnaire on an anonymous basis.

Using postal addresses provided by the Official College of Dentists of Biscay, the questionnaires were sent to all registered dentists in Biscay (except those who had explicitly stated that they did not wish to receive letters from the College). The questionnaires were sent in May 2014. We rented a post office box to receive the completed questionnaires, the deadline for their return being July 31, at which point we closed the box. A total of 931 registered dentists received the questionnaire.

The dichotomous closed questions were coded as follows: Yes, No, or Don’t know/No answer.

In open-ended questions, data analysis was performed by categorizing the answers. These categories were chosen to be broad and mutually exclusive.

Data were recorded in an Excel worksheet (Microsoft Corporation, Redmond, Washington, USA). Data analysis was carried out using SPSS for Windows, Version 15.0 (SPSS, Chicago, Illinois, USA). We performed descriptive analysis and explored differences using chi-square tests or analysis of variance (ANOVA), as appropriate, depending on nature of the data.

## Results

Out of the 931 questionnaires sent, 261 (28%) were returned completed. Among the respondents, the mean age was 44.3 years (SD=11.05) (range: 25 to 69) and the mean number of years of experience was 18.7 (SD=9) (range: 1 to 44).

**Figure 2:**
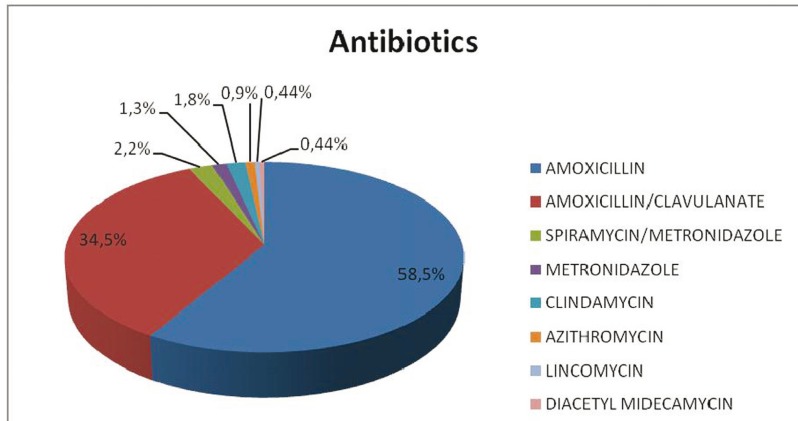
Types of antibiotics that would be prescribed for extraction of completely bone-impacted third molar, by percentage of respondents.

**Figure 3:**
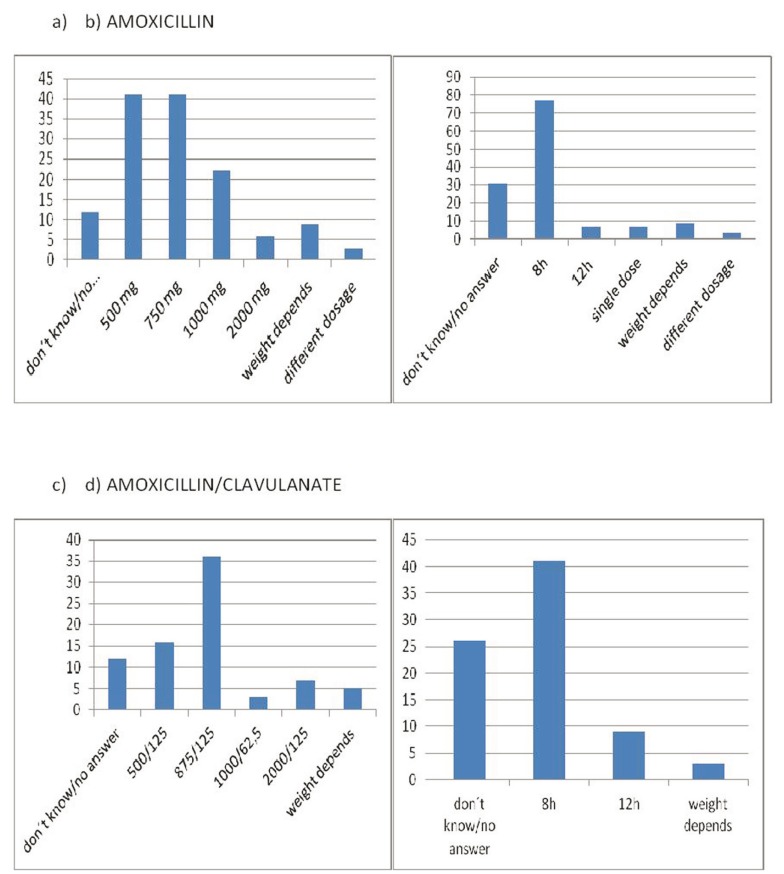
Antibiotic regimens with amoxicillin (a,b) and amoxicillin/clavulanic acid (c,d).

Considering the case presented, 83% considered it necessary to prescribe antibiotics. We did not find significant differences as a function of the age of the dentists (ANOVA *p*=0.22) or their years of professional experience (ANOVA *p*=0.45). Specifically, 58.5% said that they would prescribe amoxicillin and 34.5% amoxicillin/clavulanic acid (Fig. 2), and 60% that they would prescribe the antibiotic both before and after surgery and 4.6% only before surgery. The mean proposed duration of treatment was 7 days (SD=1.74) (range: 1 to 10). The most common regimens were amoxicillin 500 or 750 mg every 8 hours or amoxicillin/clavulanic acid 875/125 mg every 8 hours, in both cases for a mean period of 7 days (Fig. 3).

Regarding intraoperative socket irrigation, 74 (28%) of the respondents said that they would irrigate with an antiseptic containing chlorhexidine and 22 (8%) that they would do so with saline. We recorded all the products mentioned by respondents, even those with no known antiseptic properties ( Table 1). The strategy proposed was not influenced by the dentists’ age (*p*=0.16) or their years of professional experience (ANOVA, *p*=0.12).

**Table 1:**
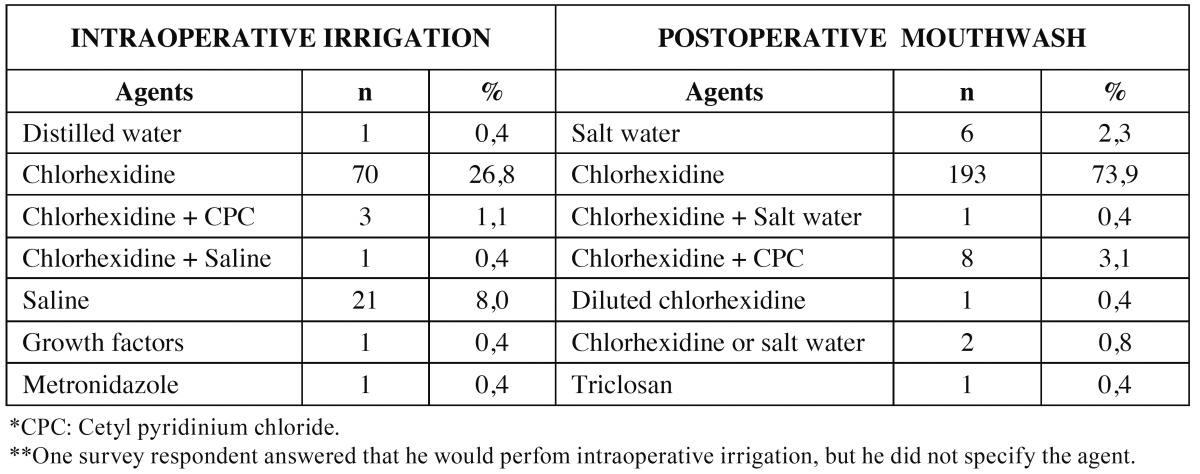
Antiseptics and other mouthwashes used for immediate post-extraction socket irrigation and postoperative treatment.

A total of 212 (81%) of respondents said that they would only prescribe antiseptics after surgery, and of these, 205 (97%) would use chlorhexidine, alone or with other agents ( Table 1). As for antibiotics and intraoperative irrigation, we did not find significant differences in the postoperative prescription of mouthwash by dentists’ age (ANOVA, *p*=0.53) or experience (ANOVA, *p*=0.62). Lastly, 69 dentists (26%) said they would prescribe chlorhexidine, alone or with other agents both intra- and post operatively.

Notably, we found a significant correlation between prescribing antibiotics and using chlorhexidine both intra- and post operatively (chi square, *p*<0.001) ( Table 2).

**Table 2:**
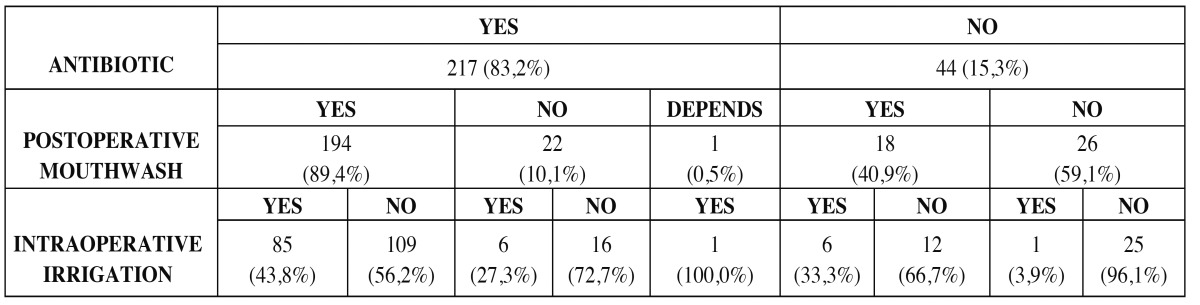
Dentists´ policy on the prescription of antibiotics and antiseptics for extraction of completely bone-impacted third molar.

## Discussion

The World Health Organization requires that patients receive medications appropriate to their clinical needs, in doses that meet their own individual requirements, for an adequate period of time, and at the lowest cost to them and their community (14). An inadequate and excessive use of medication is a poor use of resources and increases the incidence of adverse reactions. The excessive use of antibiotics is particularly serious, given that there is a progressive increase in bacterial resistance to antibiotics, and this both hampers the control of infectious diseases, and increases their clinical severity.

For some oral diseases, the scientific community accepts the use of antibiotics and this is reflected in guidelines. There is no consensus, however, on their prophylactic use in the case of tooth extraction when there is no history of infection.

Some protocols recommend antibiotic prophylaxis in healthy patients undergoing lower TME, specifically, the case of completely bone-impacted lower third molars. However, most clinical trials to assess the efficacy of preventative antibiotics in TME have not demonstrated sufficient efficacy to recommend their routine use (1-6). In the specific case of the extraction of completely bone-impacted lower third molars, a recent study (6) concluded that treatment with amoxicillin/clavulanic acid 2000/125 mg was not effective for preventing postoperative infection.

Further, a 2012 Cochrane meta-analysis (5) concluded that “due to the increasing prevalence of bacteria which are resistant to treatment by currently available antibiotics, clinicians should consider carefully whether treating 12 healthy patients with antibiotics to prevent one infection is likely to do more harm than good”.

Given all this, we wondered what attitude dentists adopt when faced with patients who require complex tooth extraction but have no history of infection. To explore this question, we designed a questionnaire that was very simple, in order that dentists would be able to reply easily, intuitively, and rapidly. Specifically, we provided general data on the case of an otherwise healthy individual, avoiding complex situations (for example, patients with cancer immunosuppression, or metabolic disorders); thereby ensuring that it was not necessary to make mathematical calculations.

A limitation of this study is the simplicity of the design of the questionnaire, but it is likely that the response rate would have been lower with a more complex questionnaire. We opted to keep the design simple, to maximize response rate and thereby obtain data as representative as possible of the opinion of dentists in Biscay. Indeed, the questionnaire response rate was higher than expected, reaching almost 30%, reflecting the opinions of 261 dentists. Further, it should be taken into account that not all dentists regularly perform lower TME, let alone in cases in which the tooth is completely covered by jaw bone.

Overall, 52.1% of respondents were under 45 years old and 45.9% were 45 to 64 years old, while 0.8% were 65 years old or above. These percentages are comparable to those reported for dentists in 2013 in the statistics on registered health professionals in Spain, where 59.6% were under 45 years old, 35.4% were 45 to 64 years old, and 5.0% were 65 years old or older. That is, our sample has similar age characteristics to dentists across Spain.

The results of the survey reflect a lack of consensus among participating dentists with respect to antibiotic prophylaxis in lower TME, but that there is a tendency to prescribe antibiotics. As many as 83% of the dentists said they would prescribe some type of antibiotics for the case presented. Most of them would use amoxicillin, alone or in combination with clavulanic acid, though there were striking differences in the antibiotic regimens proposed.

These results are consistent with those obtained in previous survey carried out in 2009 with 105 oral surgeons in Spain; all respondents said that they would prescribe antibiotics for teeth extraction surgery with osteotomy, 55% using amoxicillin and 45% amoxicillin/clavulanic acid (15). In contrast to these findings, in a survey carried out in Swiss dentists, presented with a case of partially bone-impacted lower third molar in a healthy 17-year-old woman, 81.4% said that they would not administer antibiotics prophylactically, only 18.6% saying that they would prescribed them routinely (16).

As for the antibiotic regimen, it is surprising that 18.4% of dentists in our study said they would only prescribe antibiotics after surgery, when the main objective of antibiotic prophylaxis is to achieve high blood levels of antibiotics during surgery and in the immediate postoperative period. For this, in a considerable proportion of this type of surgical interventions, it may be sufficient to only administer antibiotics before surgery, an approach considered by just 4.6% of respondents.

We also found marked differences in attitudes towards the prescription of antiseptics intra- and post operatively. A meta-analysis (11) on the use of oral antiseptics for the prevention of dry socket (alveolar osteitis), published in 2012, concluded that there is some evidence that chlorhexidine as a mouthwash or gel in the socket after tooth removal provides benefits in terms of preventing the condition. In our study, more than 80% of respondents said they would prescribe antiseptics in the postoperative period, and of these, 75% opted for chlorhexidine.

On the other hand, no clinical trials have been conducted to assess the efficacy of immediate post-extraction socket irrigation with chlorhexidine. In our case, 74 respondents reported that they would use this agent, while 22 indicated they would only irrigate with saline.

It could be supposed that dentists would prefer either prophylactic antibiotherapy or the use of antiseptics, such as chlorhexidine. However, this was not the case in our sample: those who would prescribe antibiotics also tended to prescribe chlorhexidine, while conversely, those who would not use any antibiotic prophylactically tended not to use antiseptics either, and the correlation was statistically significant.

We found that almost 8 out of 10 dentists would prescribe antibiotics and antiseptics in completely bone-impacted third TME in healthy patients, in the absence of infection. This approach is not backed by the available scientific evidence. What is more, it should be taken into account that there has been an increase in antibiotic-resistant bacteria in the oral micro flora in recent years, and this is attributable to over-prescription and/or poor patient adherence to treatment. We also should not forget that there is another risk of using antibiotics, namely, adverse reactions.

We believe that the decision to use antibiotic prophylaxis is not always based on scientific criteria. Previous experiences, beliefs, prejudice and expectations of patients are likely to influence the decision of dentists concerning antibiotic prophylaxis. Legal responsibilities may also condition dentists’ behavior, as there is no single accepted protocol.

For all these reasons, high-quality studies should be carried out to provide evidence on the effectiveness of antibiotic prophylaxis and to established protocols for cases when antibiotics should be considered necessary, specifying the most appropriate type and regimen for this kind of surgery. Further, cost-effectiveness studies should be performed to guide clinical decision making. Evidently, it would also be important to publish and disseminate the results of such research, to ensure that health professionals have and act on the basis of the best available evidence. Patient education is also a key to avoid over-prescription of antibiotics when they are not justified from a scientific point of view. In relation to this, it is very important to run public information campaigns.

Finally, it should be highlighted that there are clinical trials that seem to demonstrate the efficacy of the use of chlorhexidine for the prevention of alveolar osteitis, as well as for decreasing bacteremia and managing postoperative pain. No significant problems have been reported associated with its use, with the exception of two cases of adverse reactions (11). In this context, well-designed clinical trials to assess its efficacy, explore different regimens and monitor for adverse reactions would help us provide the best evidence-based treatment.
